# The new role of telemedicine in child psychiatry in the period of pandemic caused by spreading of the COVID-19

**DOI:** 10.1192/j.eurpsy.2021.1770

**Published:** 2021-08-13

**Authors:** L. Baranskaya

**Affiliations:** Psychiatry, Psychotherapy&narcology, Ural State Medical University, Yekaterinburg, Russian Federation

## Abstract

**Introduction:**

In the period of Covid-19 both adults and children have a great number of the most varied negative social and psychological factors.

**Objectives:**

The study of the necessity of telemedicine technologies for child and teenage psychiatric service.

**Methods:**

During two month, parents of 128 children, aged 3-18, have applied for consultative help to the Department of Psychiatry, Psychotherapy and Narcology of the Ural State Medical University.

**Results:**

In total, the number of consultations has grown by 23% compared to the same period of the previous year. The greatest number was connecting with emotional and behavioral disturbances that usually begin in childhood – 23.0%, neurotic connected with stress and somatoform disorder – 21.1%, and affective disorders –14.1%. The least amount of cases were of children and teenagers with psychiatric diagnoses: disorders of a schizophrenic character – 7% and the mentally retarded – 6.2%. These data points that the limitation of the possibility of receiving a psychiatric consultation in person did not lessen the number of instances when parents of children and teenagers applied for help. On the contrary, this period showed a growth of the number of parents who applied for help, mainly due to the increase in the proportion of anxiety and phobia disturbances, plus behavioral disruptions. At this, the number of neurotic cases connected with stress and somatoform disturbances in children increased by 92.0%, whereas emotional and behavioral disturbances that usually begin in childhood increased by 45.0%.

**Conclusions:**

Thus, telemedicine technologies in child psychiatry have proved to be an effective and necessary means.

**Disclosure:**

No significant relationships.

